# Supplementary material for: Efficient Photodegradation of Dyes from Single and Binary Aqueous Solutions Using Copper(II) Coordination Polymers
Source: Molecules. 2025 Apr 8;30(8):1652. doi: 10.3390/molecules30081652 (PMC12029952; doi:10.3390/molecules30081652)
Supplement: Supplementary file 1 [file molecules-30-01652-s001.zip › molecules-3554991-supplementary.pdf]

Supplementary File

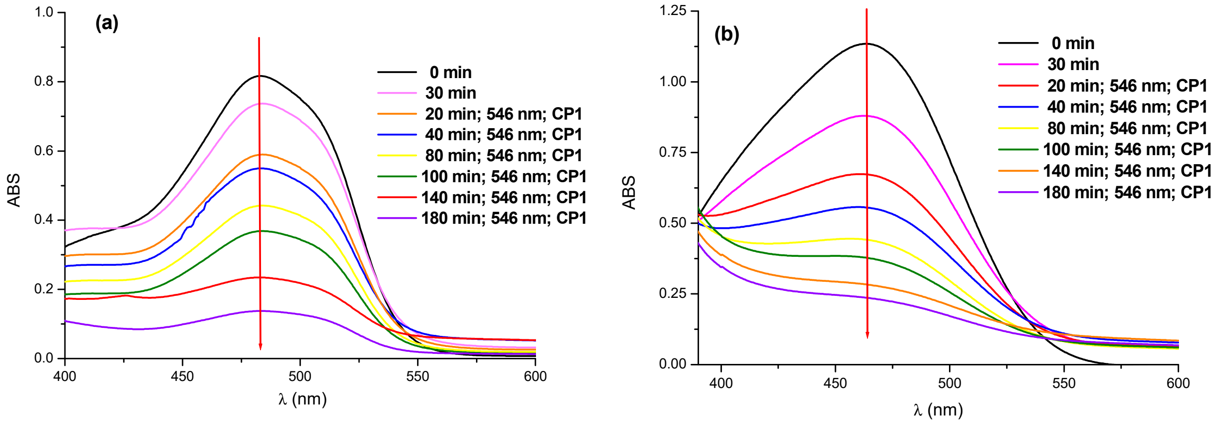

Figure. S1. Time-dependent UV-vis spectra of aqueous solutions of Acid Orange 7 (a) and Methyl Orange (b) upon irradiation with visible light using **CP1**

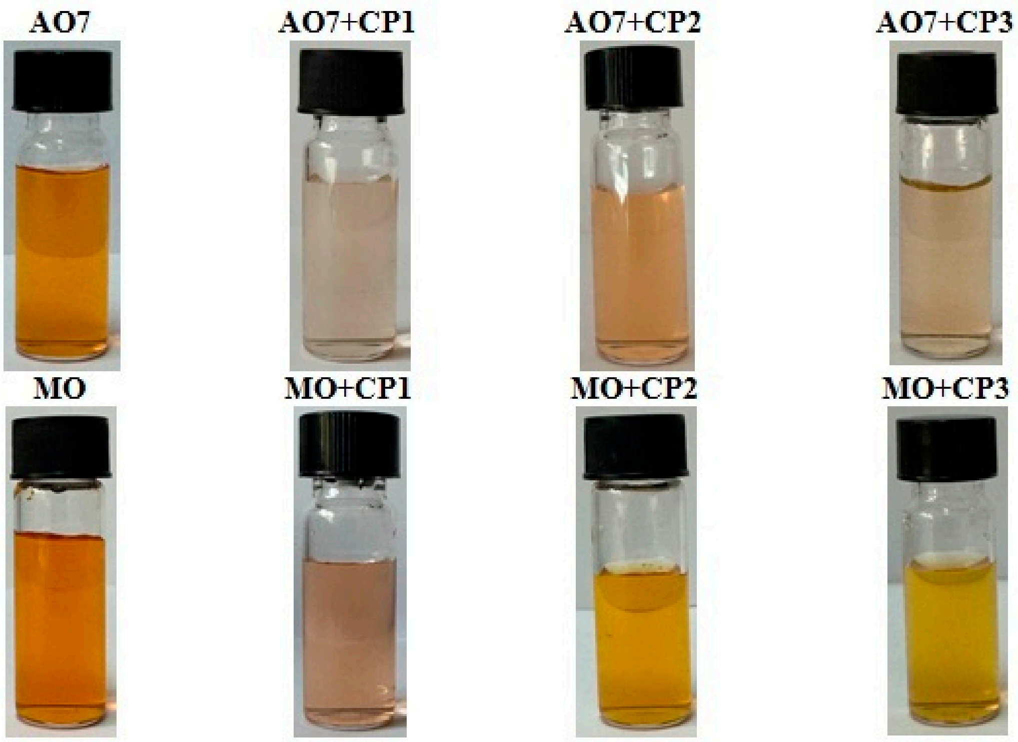

Figure. S2. Changing the color of the dye solutions over time, during the photodegradation process

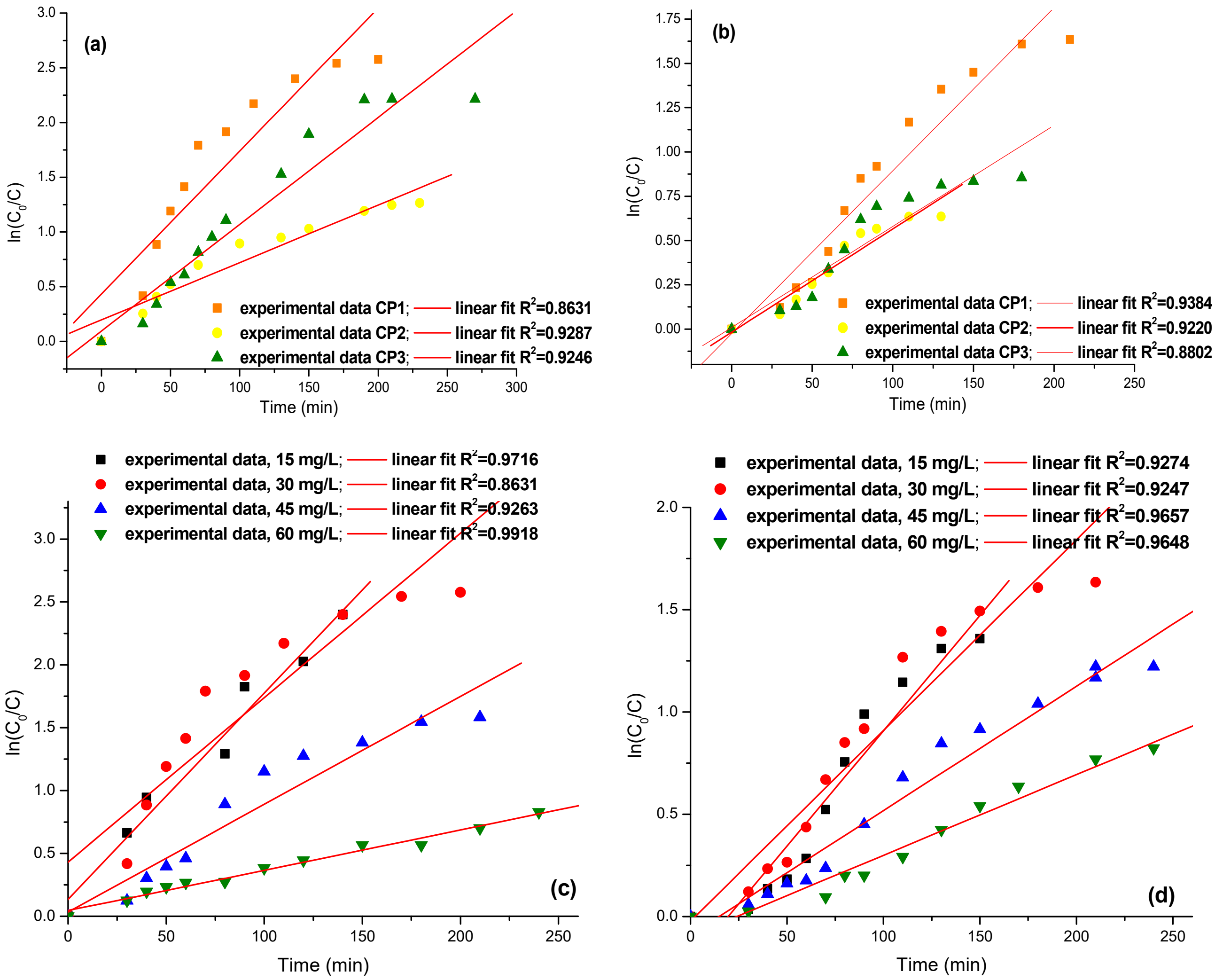

Figure. S3. Kinetics plots and the fitting curves for the photodegradation of AO7 (a, c) and MO (b, d) using **CP1**

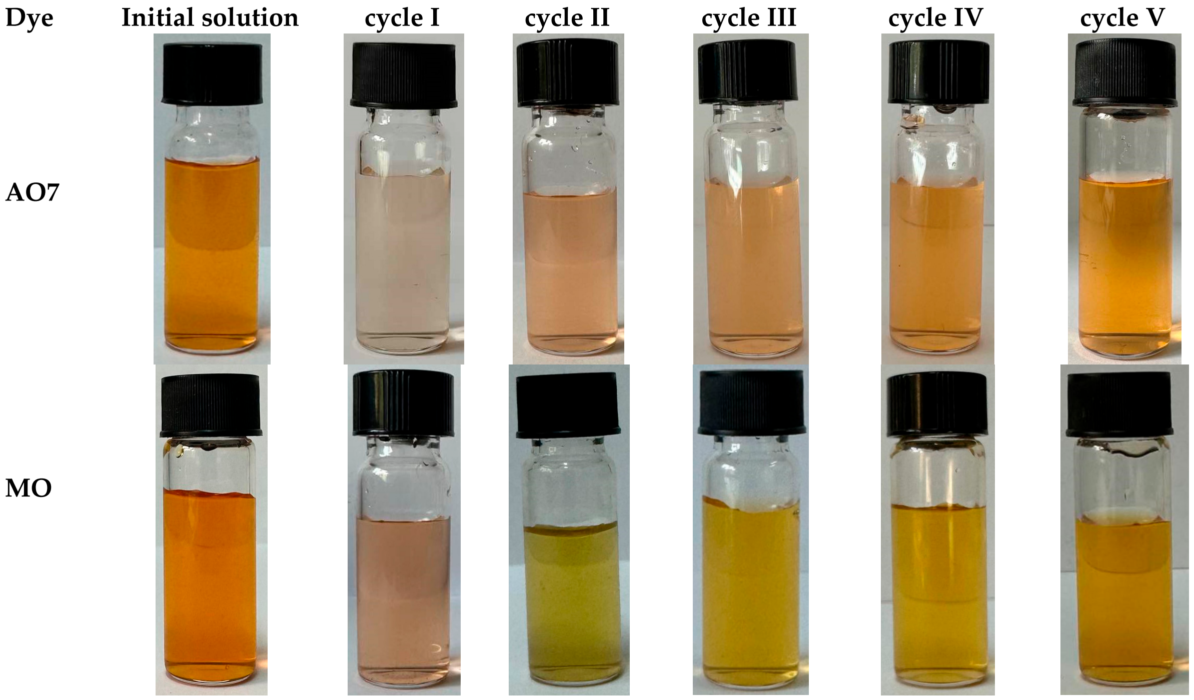

Figure. S4. Initial dye solutions and at the of the dye degradation process using **CP1**, during five photodegradation cycles

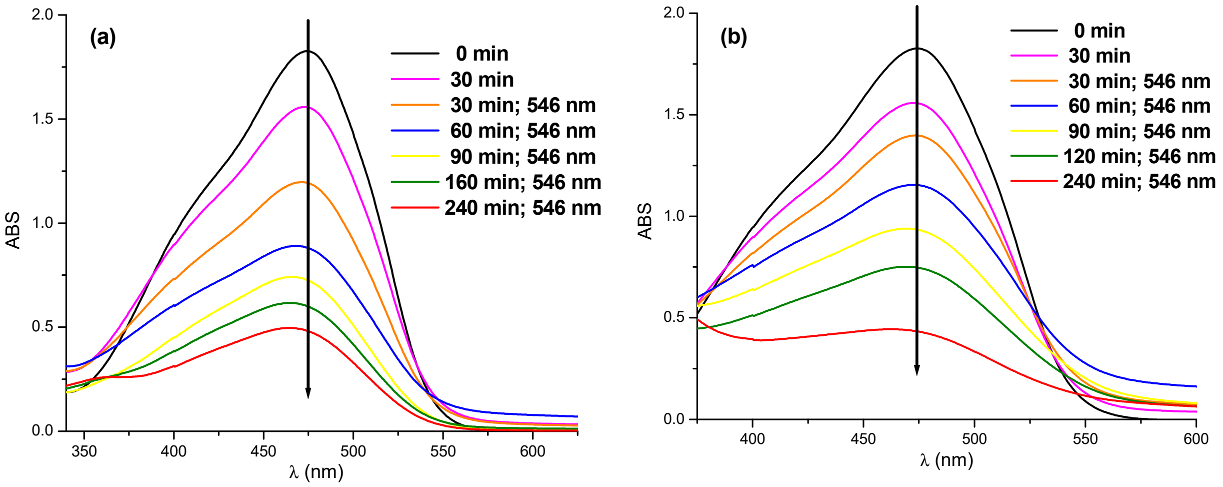

Figure. S5. Time-dependent UV-vis spectra of binary dyes mixture with (a) and without (b) adding H_2O_2

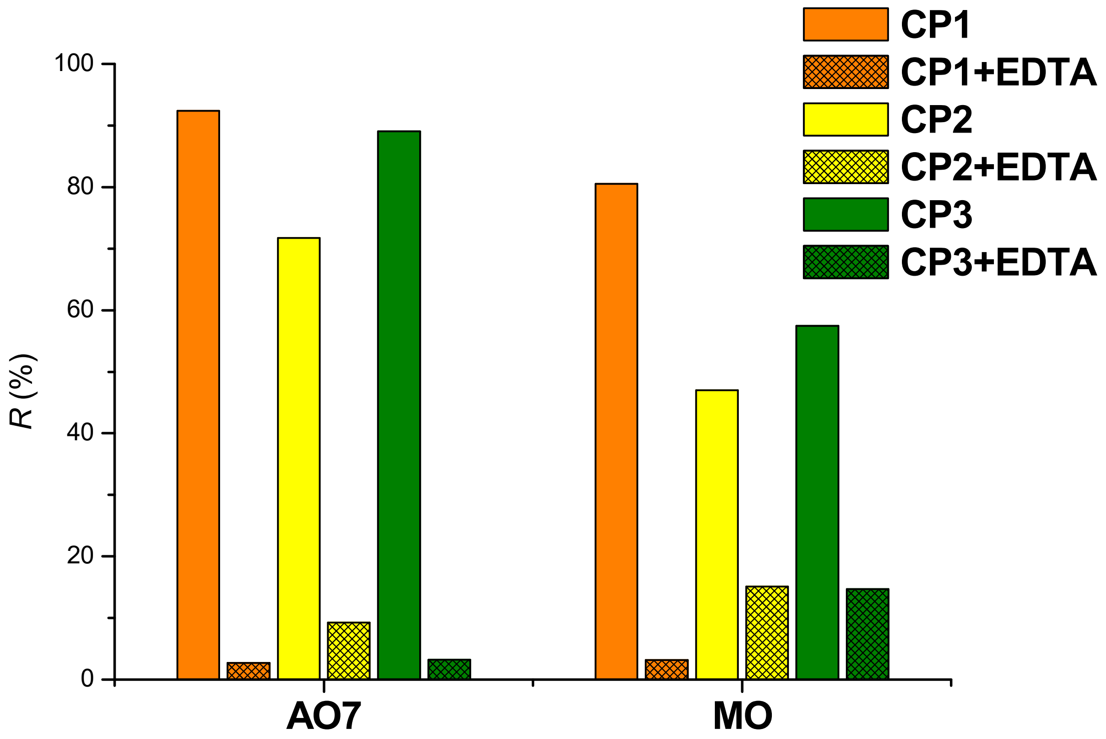

Figure. S6. Degradation efficiency of AO7 and MO dyes using **CP1-CP3,** with and without adding EDTA
